# Transmucosal fentanyl *vs* intravenous morphine in doses proportional to basal opioid regimen for episodic-breakthrough pain

**DOI:** 10.1038/sj.bjc.6603811

**Published:** 2007-05-22

**Authors:** S Mercadante, P Villari, P Ferrera, A Casuccio, S Mangione, G Intravaia

**Affiliations:** 1 Pain Relief and Palliative Care Unit, La Maddalena Cancer Center, Palermo, Italy; 2 Department of Anesthesia & Intensive Care, Palliative Medicine, University of Palermo, Palermo, Italy; 3 Department of Clinical Neuroscience, University of Palermo, Palermo, Italy

**Keywords:** intravenous morphine, OTFC, breakthrough pain, cancer pain, opioids

## Abstract

The use of supplemental doses of opioids is commonly suggested to manage breakthrough pain. A comparative study of intravenous morphine (IV-MO) and oral transmucosal fentanyl citrate (OTFC) given in doses proportional to the basal opioid regimen was performed in 25 cancer patients receiving stable opioid doses. For each episode, when it occurred and 15 and 30 min after the treatment, pain intensity and opioid-related symptoms were recorded. Fifty-three couples of breakthrough events, each treated with IV-MO and OTFC, were recorded. In episodes treated with IV-MO, pain intensity decreased from a mean of 6.9 to 3.3 and to 1.7 at T1 and T2, respectively. In episodes treated with OTFC, pain intensity decreased from a mean of 6.9 to 4.1 and to 2.4 at T1 and T2, respectively. Statistical differences between the two treatments were found at T1 (*P*=0.013), but not at T2 (*P*=0.059). Adverse effects were comparable and were not significantly related with the IV-MO and OTFC doses. Intravenous morphine and OTFC in doses proportional to the scheduled daily dose of opioids were both safe and effective, IV-MO having a shorter onset than OTFC. Future comparative studies with appropriate design should compare titration methods and proportional methods of OTFC dosing.

Patients with chronic cancer pain often report wide fluctuations in pain intensity. In the cancer population, breakthrough pain (or episodic pain) is a transitory flare of pain superimposed on an otherwise stable pain pattern in patients treated with opioids (Portenoy *et al*, 1999a). The availability of supplemental doses of opioids (rescue medication) in addition to the continuous analgesic medication is the main treatment suggested to manage these pain flares. Current dosing recommendations for breakthrough pain generally suggest that the effective dose of breakthrough pain medication must be a percentage of a patient's total daily opioid dose (Hanks *et al*, 2001). These recommendations, which are based entirely on anecdotal experience, favour the selection of a short-acting opioid at a dose proportionate to the total daily dose. However, an oral dose form can take a longer time to relieve pain. As pain relief is usually required urgently, routes of administration designed to deliver drugs rapidly are often chosen. A short onset of effect is commonly obtainable only with parenteral or transmucosal administration of opioid analgesics.

Intravenous morphine (IV-MO) has been found to be highly effective and safe, as only a low intensity of opioid-induced adverse effects was observed, even when administering large doses (Mercadante *et al*, 2004). While the intravenous route for morphine administration is feasible in acute units, in some other centres is not favourite, and at home injections are not easily manageable.

On the other hand, studies of oral transmucosal fentanyl citrate (OTFC) have shown that this approach produces a faster onset of relief and a greater degree of pain relief than the oral morphine, at 15, 30, and 60 min (Christie *et al*, 1998; Farrar *et al*, 1998; Portenoy *et al*, 1999b; Coluzzi *et al*, 2001). A lack of relationship between the effective OTFC dose and the fixed schedule opioid regimen, regardless of the opioid used, was observed suggesting the need to titrate the dose of OTFC. This observation contradicted the anecdotal assumption that the effective dose as needed is a percentage of the opioid daily dose. The reasons for the above finding have not been clearly explained. This makes the practical use of OTFC in the raw clinical setting, or at home, difficult. Moreover, using different pieces of OTFC for treating each episode may be time consuming exceeding the spontaneous duration for breakthrough pain, which can spontaneously subside, as evidenced by placebo-treated patients.

From the practical point of view, in our daily activity, most patients have been reluctant to try the dose, either on in-patient or outpatient basis, and avoid using the OTFC, preferring, at the end, traditional oral dosing of morphine. The aim of this randomised, crossover, controlled study was to verify the safety and effectiveness of a fixed dose of OTFC, proportional to the daily dose, compared to the standard treatment available in the unit, which is IV-MO, used in a similar way for the management of breakthrough pain.

## PATIENTS AND METHODS

This is a randomised, crossover, controlled study comparing the effectiveness of a fixed dose of OTFC and IV-MO, for the management of breakthrough pain. A consecutive sample of 105 cancer patients, who were admitted to a Pain Relief and Palliative Care unit for a period of 1 year, receiving a stable opioid dose of oral morphine or transdermal fentanyl for their cancer-related pain reporting an acceptable pain relief, was screened for the study. Ethical committee approval was obtained.

### Study population

Adult patients with cancer-related pain were eligible if they were receiving opioids regularly at doses of more than 60 mg of oral morphine equivalents, had an acceptable pain relief, and presented no more than two pain flares per day. Patients were excluded from the study if they had important metabolic alterations, cognitive failure, lack of cooperation, or extreme ages (under 18 and over 80 years). Patients with predominant incident pain or with short-lived episodes of neuropathic pain resolving spontaneously in few minutes were also excluded. Finally, patients receiving oral morphine equivalents in a dose range of 270–330 mg (see below), and patients who presented more than two episodes or just one episode during the daily hours (from 0700 to 1900), or pain flares repeating after with short intervals (less than 6 h) were also excluded (see the protocol described below).

Forty patients met the inclusion criteria and gave their informed consent. According to the department policy, an intravenous line was established for emergency treatment of symptoms. Increases of pain intensity were considered only if patients felt that there was a clear difference with basal pain and required a medication as needed. Patients were encouraged to call when their pain got severe.

### Procedures

Patients were planned to receive IV-MO and OTFC for each couple of breakthrough pain events. The order of administration was computer-generated to produce equivalent sequence orders, 50–50%, and the wash out period between the pain flares was at least 6 h. Patients who repeated the sequence on another day received the opposite sequence. Only patients presenting a couple of episodes occurring between 0700 and 1900 were taken into consideration. Thus, patients received both treatments, serving as their own control. Patients who presented more than two episodes or just one episode during the daily hours (from 0700 to 1900) were excluded, so that only days in which two episodes occurred within this interval of 12 h were considered, to assure the presence of physicians in the unit, and minimise the occurrence of possible complications.

The rationale in choosing the doses to be administered was based on a previous experience with IV-MO in doses of 4 mg in patients receiving an equivalent dose of oral morphine of 60 mg (Mercadante *et al*, 2004), and on the suggestion from previous studies of OTFC that 200 *μ*g OTFC should be administered in patients receiving at least 60 mg day^−1^ of oral morphine or 25 *μ*g h^−1^ (0.6 mg day^−1^) of transdermal fentanyl (Christie *et al*, 1998; Farrar *et al*, 1998; Portenoy *et al*, 1999b; Coluzzi *et al*, 2001). This does not mean that the dose of the two drugs is equianalgesic, despite some information drawn from previous local experience and studies performed in other settings (Lichtor *et al*, 1999).

The IV-MO dose was administered for about 5 min at 1/5 of the oral daily dose, converted using an equianalgesic ratio of 1/3 (IV/oral) (Mercadante *et al*, 2004). For example, a daily morphine oral dose of 60 mg corresponds to an intravenous dose of 20 mg (1/3 ratio), and then is converted to 4 mg (20%), to be used as the dose for episodic pain. Written orders were given and IV-MO was administered by nurses, already trained in using such an approach, as a standard practice of the unit. Oral transmucosal fentanyl citrate was administered, after a previous explanation of the use, by patients themselves, also assisted by nurse, in doses proportional to the basal daily opioid dose, as described for IV-MO. Doses were rounded off to the closest value for patients receiving intermediate dosages. Only one stick of OTFC was used. Thus, patients receiving oral morphine equivalents in the dose range of 270–330 mg were excluded, owing to unavailability of the calculated dose of OTFC (1000 *μ*g).

For each episode, when it occurred (T0), 15 min after (T1), and 30 min after (T2) the study of drug administration, patients were asked about the following parameters, which were recorded: pain intensity, using a numerical scale from 0 to 10, and opioid-related symptoms, using a scale from 0 to 3 (absent, slight, moderate, severe). These scales have been reported in several previous papers on same subject (Mercadante *et al*, 2004, 2005, 2006), are familiar to the staff, and represent the standard in the unit. Data were collected by nurses trained in symptom measurement as a part of their daily activity. They were also previously trained for data collection of breakthrough pain events. This tertiary unit offers a high level of nurse training and monitoring (Mercadante *et al*, 2003; Fine, 2005). In particular, for each episode, nurses were instructed to routinely collect changes in pain intensity (numerical scale 0–10) and emerging problems: when they are called for pain increases considered to be severe in intensity by patients (T0), 15 min after IV-MO injection (T1) and 30 after (T2).

Daily doses of oral morphine equivalents and basal pain intensity were also recorded. A decrease in pain intensity of at least >33% at T1, not requiring further treatment for the next 2 h, was considered as an effective treatment of each episode. Patients were offered the common prescription used in the unit, that is IV-MO, but at half dose, if they were not satisfied with the treatments within T1.

### Data analysis

A power analysis indicated that a sample size of 25 episodes per group would allow to detect a 20% difference (*P*<0.05, power=0.8). This computation assumes that the mean difference is 0.20 with a 95% confidence interval (CI) of 0.07–0.33 and the common within-group standard deviation of 0.28.

Frequency analysis was performed with *χ*^2^ test. The univariate and multivariate repeated measures analysis (analysis of variance (ANOVA)) was used to compare the scores or the means of non-parametric and parametric variables, respectively, at different time intervals. The one-way ANOVA and Mann–Whitney *U* statistic test were used to compare the different parametric or non-parametric variables. All *P*-values were two-sided and *P*-values less than 0.05 were considered statistically significant.

## RESULTS

**Figure 1:**
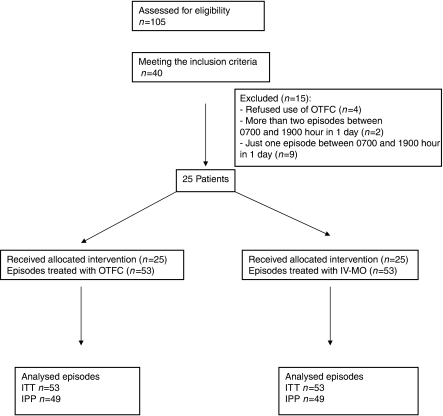
CONSORT flowchart.

Patients' characteristics are listed in Tables 1 and 2. All patients had their basal pain under control (mean 2.9, 95% CI 2.3–3.6). Fifteen patients were excluded from the study: four patients were unable to use OTFC, two patients had more than two episodes between 0700 and 1900, nine patients had just one episode to treat (Figure 1).

Twenty-five patients completed 53 couples of breakthrough events (a mean of 2.12 couple of episodes for each patient, recorded on different days), each randomly treated with IV-MO and OTFC, during admission. A total of 25 couples were IV-MO/OTFC sequences, and 28 couples were OTFC/IV-MO sequences.

In episodes treated with IV-MO, pain intensity decreased from 6.9 (95% CI 6.6–7.2) to 3.3 (95% CI 2.7–3.8) and 1.7 (95% CI 1.2–2.3) at T1 and T2, respectively. This reduction was more than 33% in 39 (74%) and in 46 episodes (87%) at T1 and T2, respectively, and more than 50% in 29 (55%) and in 40 episodes (75%), at T1 and T2, respectively.

In episodes treated with OTFC, pain intensity decreased from 6.9 (95% CI 6.6–7.2) to 4.1 (95% CI 3.6–4.7) and 2.4 (95% CI 1.8–2.9) at T1 and T2, respectively. This reduction was more than 33% in 30 (57%) and 45 episodes (85%) at T1 and T2, respectively, and more than 50% in 20 (38%) and in 40 episodes (75%) at T1 and T2, respectively.

A statistical difference between the two treatments was found at T1 (*P*=0.013, univariate repeated measures analysis ANOVA), whereas at T2 the difference did not attain a statistical significance (*P*=0.059, univariate repeated measures analysis ANOVA). Three patients and one patient who had received OTFC and IV-MO, respectively, required an additional (half) dose of IV-MO, as a further rescue dose. When excluding these patients with the intention for protocol analysis, a statistical difference was similarly found at T1 (*P*=0.049), but not at T2 (*P*=0.124).

At T1, a decrease of 41.4 and 51.7% in pain intensity was observed after OTFC and IV-MO, respectively (*P*=0.026). At T2, a decrease of 65.9 and 73.8% in pain intensity was recorded after OTFC and IV-MO, respectively (*P*=0.136). No differences between the two groups were observed in the number of episodes with a reduction of more than 33 and 50% at T1 (*P*=0.66 and 0.39) and T2 (*P*=0.23 and 0.20), respectively.

The outcome was not related to the basal regimen, and as a consequence to IV-MO and OTFC doses. Statistical tests with exact critical values were performed to evaluate differential carryover effect and period effect between treatments. There were no statistically significant period or carryover effects (*P*=0.82 and 0.19, respectively).

No differences in age, gender, pain mechanism, time of events, or kind of opioid used as basal regimen were found.

### Adverse effects

Acute adverse effects occurring after IV-MO and OTFC were comparable and corresponded to those commonly observed with opioid therapy. In most patients, the level of adverse effects after the study medication was undistinguishable from that owing to basal opioid analgesia. A minority of episodes were followed by adverse effects with a certain intensity (2/3 on the scale used). Moderate adverse effects in episodes treated with OTFC were: nausea (four episodes), drowsiness (seven episodes), and confusion (one episode). Moderate adverse effects in episodes treated with IV-MO were: nausea (two episodes), drowsiness (10 episodes), confusion (three episodes) (see Table 3). No severe adverse effect was recorded. The occurrence of such adverse effects was not related with the basal dose and, as a consequence, with the IV-MO or OTFC doses given for breakthrough pain.

## DISCUSSION

Despite the growing interest on breakthrough or episodic pain in the last 10 years, few prospective comparison studies have been performed. Oral transmucosal fentanyl citrate was compared with the standard treatment used in the unit, which is an intensive acute pain relief and palliative care unit, where strict monitoring is essential and an intravenous line is commonly inserted to achieve rapidly pain and symptom control. We have also chosen IV-MO as a control, as this modality could better fit the onset of OTFC for comparison, although the use of IV-MO is often restricted to selected palliative care units. While subcutaneous route is more frequently used in the setting of hospice care, probably, it would result in an expected delay in the onset of the effect in comparison with OTFC.

In this comparative trial, although both treatments were effective, IV-MO had a shorter onset of analgesia in comparison with OTFC, while producing similar adverse effect profile, demonstrating safety and effectiveness. A more important information could be gathered by this study. Oral transmucosal fentanyl citrate, given in doses proportional to the basal opioid regimen, was quite effective and, above all, safe, avoiding to titrate the dose, which is unsuitable for some patients, reducing their compliance with the treatment. In patients receiving 180 mg of oral morphine equivalents or more, 39 episodes (73.5%) were treated with doses of 600 *μ*g of OTFC or more in the first instance, producing acceptable adverse effects, which occurred equally, independently of the dose. This observation contrasts with almost all studies of OTFC (Christie *et al*, 1998; Farrar *et al*, 1998; Portenoy *et al*, 1999b; Coluzzi *et al*, 2001) , where the principal finding was a lack of correlation of the OTFC dose with the basal opioid regimen, although some data could be interpreted in another way. For example, many patients on higher doses of original medication generally required larger doses of OTFC and in successful patients the regular rescue dose was a moderate predictor of the effective OTFC dose. In one of the controlled studies of OTFC, a relationship between the OTFC dose and the fixed scheduled opioid had been already found, and regular rescue dose was a moderate predictor of the effective OTFC dose. However, only 19% of the variability of the final dose of OTFC was explained by basal doses of opioids, according to the low-R-square vale of the model used (Christie *et al*, 1998). Recent observations from data pooled from trials of OTFC showed a statistically significant relationship between the breakthrough dose and the around-the-clock dose, despite an enormous interindividual variability in patients' dose requirements for breakthrough pain (Hagen *et al*, 2007).

The aim of the present study was not to compare effective titrated doses with doses proportional to the scheduled daily doses, but two fast methods for treating breakthrough pain were compared, using a similar approach that is providing opioids at a dose proportional to the basal regimen.

Some issues gathered by these previous studies should be pointed out. As 66% of the episodes treated with placebo did not require an additional dose of medication, the episodes recorded were possibly short-lived or not too much severe, and possibly spontaneously resolved. This can also explain why minimal doses of oral morphine equivalents (about 20 mg) were effective in patients taking a mean basal dose of 100 *μ*g per hour of fentanyl (about 240 mg daily of oral morphine doses equivalents). Eligible patients were defined as having their basal pain no more than moderate, and presenting no more than four episodes per day. On the other hand, some patients could not be universally considered (mean intensity of 4.7–4.8 of basal pain, with some patients at the highest extremes) as having well-controlled pain, especially if matched with a pain intensity of breakthrough events of 6.8 on average (with some patients at the lowest extremes). No distinction was made between incident pain, dependent on activity, and other mechanisms, which can have a different temporal pattern. Finally, almost no adverse effects were reported with usual breakthrough medication in comparison with OTFC, which doses were titrated in patients apparently responsive to their usual medication, suggesting that probably most patients were undertreated either with basal or ‘as needed’ medication.

Of interest, in previous studies of OTFC, up to four episodes per day were treated, suggesting that the treatment was sometime repeated more frequently than every 6 h. Six hours should be a sufficient interval to avoid overlapping of effects between OTFC and IV-MO administration and vice versa. On the other hand, the increase in pain intensity, typical of the breakthrough event, which triggers drug administration, gives evidence that the effect of the previous medication is evanished.

In this study, criteria for selecting breakthrough events were strict. Adequate pain levels (about 3/10 intensity) and severe pain intensity of the episodes (about 7/10 intensity) were chosen to make a clear distinction between basal pain intensity and pain flare intensity. We also selected patients having no more than two episodes per day, to avoid possible inclusion of patients with disputable pain control. Specifically, for inclusion, we have chosen only days when both randomised treatments were used, facilitating also patients' compliance for comparison, but at a distance of at least 6 h, to avoid overlapping effects. Given that the study was not blinded, a learning effect in using OTFC cannot be excluded in subsequent administrations, but this was inevitable. However, no particular differences between subjects who received one or more OTFC treatments were found. When evaluating differential carryover effect and period effect between treatments, there were no statistically significant period or carryover effects. Of interest, previous studies did not take into consideration an acceptable time interval between events (Christie *et al*, 1998; Farrar *et al*, 1998; Portenoy *et al*, 1999b; Coluzzi *et al*, 2001), included in the present protocol, and comparison could have paradoxically been inferred by titration to find an adequate dose and previous selection of responsive patients to both previous opioids and OTFC (exclusion of patient owing to adverse effects during open-label titration).

While diminishing the recruitment power, this method was more selective. Moreover a fixed time for response evaluation was taken into consideration, as this is an acceptable burden and timing for patients. Finally, patients who presented clear characteristics of incident pain due to movement were excluded. The analgesic treatment of this kind of pain is more difficult to assess, owing to the predictability of the event and variability in patients' behaviour, which can confound the outcome.

To evaluate the effectiveness of a treatment of a 50% cutoff point for the percentage of maximum pain relief is considered as a consistent clinical end point of pain half relieved, easily understood by professionals and patients. However, the minimal end point to efficacy has been found to be a decrease in pain intensity of at least >33%, 15 min after the study medications. This level of change in pain intensity has been best associated with clinically important differences by patients (Farrar *et al*, 2001). As expected, OTFC has been found to be more effective and rapid than oral morphine for treating breakthrough pain, producing a >33% change in about 42% of patients within 15 min after administration (about 32%) (Coluzzi *et al*, 2001). In an open-label study, this level was achieved in about 15 min in more than 90% of events treated by IV-MO, despite selection criteria were more strict and included episodes with a high intensity (more of 7/10 on a numerical scale) (Mercadante *et al*, 2004). This finding was confirmed in the present study, as IV-MO provided clinical differences in about 75% of treated episodes 15 min after injection. Of interest, OTFC, used in doses proportional to the basal regimen, was effective in about 57% of treated episodes, a percentage higher than that reported in previous studies (Christie *et al*, 1998; Farrar *et al*, 1998; Portenoy *et al*, 1999b; Coluzzi *et al*, 2001).

To test the efficacy of IV-MO and OTFC, two similar episodes occurring in the same day were selected to make the comparison easy. In postoperative studies, it has been calculated that OTFC:IV-MO ratio is 1 : 10–20 (Lichtor *et al*, 1999; Lu and Bailey, 2003). However, this group of patients were opioid-naive, and no comparison can be carried out with the present results. A ratio of about 1 : 20 was used in this study (a dose of 200 *μ*g of OTFC fentanyl corresponded to 4 mg of IV-MO) after a preliminary testing, also considering that in previous studies the starting dose of OTFC for patients receiving equivalent doses of oral morphine was 200 *μ*g (Christie *et al*, 1998; Farrar *et al*, 1998; Portenoy *et al*, 1999b; Coluzzi *et al*, 2001). As with IV-MO, OTFC doses should not exceed 20% of patients' around-the-clock medication, which should be at least 60 mg of oral morphine equivalents. For several reasons, it is unlikely that an equivalency approach is feasible under the circumstances of this study. While the availability of IV-MO is total and then predictable, individual differences in OTFC availability are likely, generally reduced in some patients, who may not use correctly the stick or may have mucosal damage, limiting absorption, although this process was monitored by nurses by direct vision. Thus, while underdosing may occur in some patients, producing a more limited effect than expected, higher availability and then more absorption and overdosing with OTFC are unlikely. This may confirm the safety of the approach used, avoiding to start titration with minimal doses of OTFC patients who are receiving high doses of opioids regularly. This practice may discourage patients, particularly outpatients, to continue titration in daily practice.

It is likely that the use of a different ratio, for example 1 : 10, would have affected (improved) the efficacy of OTFC compared to IV-MO. This means that OTFC doses of 400 *μ*g should be started in patients receiving oral morphine equivalents of 60 mg day^−1^. However, according to this protocol, 1600 or 3200 *μ*g should be administered in patients receiving 240 or 480 mg day^−1^ of oral morphine equivalents, respectively. Given the exploratory nature of this study, a more prudent ratio was used. Of interest, intensity of adverse effects were mild and acceptable in most cases, with a rate that was similar to that reported in an open long-term study where OTFC doses were titrated previously (Hanks *et al*, 2004).

Owing to lack of blindness of the study, the results should be interpreted carefully. No placebo control was considered for such patients requiring immediate pain relief and having available effective medication, and who were considered not amenable ethically with placebo, also given the exploratory use of OTFC at doses equivalent to the basal opioid regimen, never tested in previous OTFC studies where no correlations were found. The double-dummy technique requiring simultaneous treatments was unfeasible in this clinical context. The low number of patients is of concern, and probably owing to dropout rate. However, the strict selection criteria adopted (number and intensity of episodes, excluding pain on movement, type of opioid used for basal analgesia, no previous selection for adverse effects during a titration phase) should limit this bias. In any case, the meaning of this study should be interpreted as a preliminary experience for evaluating the feasibility of such an approach, rather than proposing a standard treatment, particularly given the peculiarity of the setting where the study was performed. To collect such acute data in real time, it was preferred to use very simple tools and specific time intervals, avoiding other instruments, such as pain relief intensity, which would have confused the monitoring, introducing a further burden for patients. Fifteen minutes was considered an acceptable interval to evaluate the treatment of pain flares. Another possible concern is about the use of different opioids as basal medication, which were compared in an equianalgesic range. In previous studies and a subsequent experience, this problem did not arise specific problems in response to OTFC or IV-MO (Mercadante *et al*, 2004; Farrar *et al*, 1998; Portenoy *et al*, 1999b; Coluzzi *et al*, 2001).

In conclusion, IV-MO and OTFC were equally effective for treating breakthrough pain episodes, the effect of IV-MO being faster. As IV-MO, OTFC used at a dose proportional to the basal opioid regimen was safe and effective in the majority of patients experiencing pain exacerbation, confirming figures reported previously, but contradicting previous OTFC studies. Adverse effects were compatible and not troublesome. Should data regarding the risks confirmed in a larger number of patients, this treatment could be feasible even for outpatients or home patients, without requiring complex titration procedures.

## Figures and Tables

**Table 1:** Characteristics of patients

No. of patients	25
Age	59 (95% CI 55–63)
Gender (M/F)	12/13
	
*Pain characteristics*	
Somatic	9
Somatic–visceral	3
Somatic–neuropathic	3
Visceral	4
Visceral–neuropathic	2
Neuropathic	4
	
Basal morphine dose	120 mg (95% CI 96–144)
	
*OTFC/IV-MO*	
200/4	Six patients available for nine drug sequences (in different days)=1.5 sequence for patient
400/8	Three patients available for five drug sequences (in different days)=1.6 sequence for patient
600/12	Five patients available for 14 drug sequences (in different days)=2.8 sequences for patient
800/16	One patient available for six drug sequences (in different days)=6 sequences for patient
1200/24	Eight patients available for 13 drug sequences (in different days)=1.62 sequences per patient
1600/32	Two patients available for six drug sequences (in different days)=3 sequences per patient

CI=confidence interval; IV-MO=intravenous-morphine; OTFC=oral transmucosal fentanyl citrate.

Age (mean), gender, pain mechanisms, basal oral morphine equivalent doses (mean and 95% CI). Doses of OTFC (*μ*g) and IV-MO (mg), number of patients and number of sequences for each patient for any dose level of OTFC and IV-MO.

**Table 2:** Changes of pain intensity 15 and 30 min after treatment in all patients, and in patients using different ranges of basal opioid doses (<120, 120–270, and >330 mg of oral morphine equivalents, respectively)

	**T0**	**T1**	**T2**
OTFC	6.9 (6.6–7.2)	4.1 (3.5–4.7)	2.4 (1.8–2.9)
IV-MO	6.9 (6.6–7.2)	3.3 (2.7–3.8)^*^	1.7 (1.2–2.3)^**^
			
*⩽120 mg of oral morphine equivalents*			
9 patients/14 events			
OTFC	6.1 (5.4–6.7)	3.7 (2.5–4.8)	2.4 (1.0–3.8)
IV-MO	6.3 (5.8–6.8)	3.2 (2.2–4.3)	1.7 (0.5–3.0)
			
*>120–270 mg of oral morphine equivalents*			
14 patients/33 events			
OTFC	7.2 (6.8–7.5)	4.5 (3.7–5.3)	2.6 (1.8–3.3)
IV-MO	7.1 (6.6–7.5)	3.5 (2.8–4.2)	1.9 (1.2–2.7)
			
*>330 mg of oral morphine equivalents*			
2 patients/6 events			
OTFC	7.1 (6.6–7.6)	3.7 (2.3–5.1)	1.9 (0.6–3.2)
IV-MO	7.1 (6.3–7.8)	2.7 (1.4–4.1)	1.2 (0–2.6)

IV-MO=intravenous-morphine; OTFC=oral transmucosal fentanyl citrate.

Data are expressed as mean (95% CI). ^*^P=0.013 and ^**^P=0.059 (univariate repeated measures analysis ANOVA, F=6.694 and 3.732, respectively).

**Table 3:** Adverse effects with an intensity of 2/3 on the scale used (moderate intensity) in episodes treated with OTFC and IV-MO

	**OTFC**	**IV-MO**
Nausea	4 (7.5%)	2 (3.7%)
Drowsiness	7 (13.2%)	10 (18.8%)
Confusion	1 (1.8%)	3 (5.6%)

IV-MO=intravenous-morphine; OTFC=oral transmucosal fentanyl citrate.
